# Safety and Feasibility of Video-Assisted Thoracoscopic Day Surgery and Inpatient Surgery in Patients With Non-small Cell Lung Cancer: A Single-Center Retrospective Cohort Study

**DOI:** 10.3389/fsurg.2021.779889

**Published:** 2021-11-17

**Authors:** Yingxian Dong, Cheng Shen, Yan Wang, Kun Zhou, Jue Li, Shuai Chang, Hongsheng Ma, Guowei Che

**Affiliations:** ^1^Department of Thoracic Surgery, West-China Hospital, Sichuan University, Chengdu, China; ^2^Day Surgery Center, West China Hospital, Sichuan University, Chengdu, China

**Keywords:** day surgery, enhanced recovery after surgery (ERAS), non-small cell lung cancer (NSCLC), video-assisted thoracic surgery (VATS), minimally invasive surgery

## Abstract

**Background and Objective:** This study was undertaken to evaluate how safe and viable the use of video-assisted thoracoscopic day surgery (VATDS) is for individuals diagnosed with early-stage non-small cell lung cancer (NSCLC).

**Methods:** Data obtained from the selected patients with NSCLC who underwent video-assisted thoracoscopic surgery (VATS) in the same medical group were analyzed and a single-center, propensity-matched cohort study was performed. In total, 353 individuals were included after propensity score matching (PSM) with 136 individuals in the day surgery group (DSG) and 217 individuals in the inpatient surgery group (ISG).

**Results:** The 24-h discharge rate in the DSG was 93.38% (127/136). With respect to the postoperative complications (PPCs), no difference between the two groups was found (DSG vs. ISG: 11.76 vs. 11.52%, *p* = 0.933). In the DSG, a shorter length of stay (LOS) after surgery (1.47 ± 1.09 vs. 2.72 ± 1.28 days, *p* < 0.001) and reduced drainage time (8.45 ± 3.35 vs. 24.11 ± 5.23 h, *p* < 0.001) were found, while the drainage volume per hour (mL/h) was not notably divergent between the relevant groups (*p* = 0.312). No difference was observed in the cost of equipment and materials between the two groups (*p* = 0.333). However, the average hospital cost and drug cost of the DSG were significantly lower than those of the ISG (*p* < 0.001).

**Conclusion:** The study indicated that the implementation of VATDS showed no difference in PPCs, but resulted in shorter in-hospital stays, shorter drainage times, and lower hospital costs than inpatient surgery. These results indicate the safety and feasibility of VATDS for a group of highly selected patients with early-stage NSCLC.

## Introduction

Enhanced recovery after surgery (ERAS) refers to a set of guidelines, predetermined activities, and protocols that aim to enhance clinical outcomes and costs (1). ERAS in thoracic surgery has developed to a greater degree, especially in the management of perioperative care for patients, which, combined with minimally invasive surgery, has reduced the occurrence of perioperative complications and reduced the length of stay (LOS) (2, 3). In addition, with the help of low-dose CT (LDCT), small pulmonary nodules are found in an increasing number of young people, leading to an increasing number of the patients with lung cancer being screened at an early stage (4). Therefore, better clinical decisions for these people are needed. With the development of anesthesia and surgical technology, day surgery is currently a relatively safe, economical medical model with high satisfaction for the patients. It is commonly practiced in more developed countries such as the United States and countries in Europe (5, 6). Additionally, in China, there has been notable development in day surgery practice (7). Day surgery refers to a surgical procedure that is planned and conducted with the patient discharged by the end of the day (the Chinese Ambulatory Surgery Alliance). This definition excludes outpatient surgery. In the UK, day surgery is defined as a type of surgery that is planned and performed; thereafter, the patient is discharged within a day and can be allowed to spend a day at the hospital for recovery (8). Day surgery is not a specific type of surgical procedure, but a form of healthcare pathway management. Thus, there are increased chances of success of video-assisted thoracoscopic day surgery (VATDS) for the patients with lung cancer in the early stage when ERAS is applied. However, previous research studies regarding same day surgery as a mode of thoracic surgery have been scarce (9–11). In this study, we reviewed the experience of the patients who underwent VATDS at our hospital's Day Surgery Center between June 2019 and December 2020. To determine how safe and viable that it is to use VATDS, we used propensity score matching (PSM) (12) to compare differences between patients with non-small cell lung cancer (NSCLC) receiving video-assisted thoracic surgery (VATS) in the thoracic day surgery group (DSG) or the inpatient surgery group (ISG).

## Patients and Methods

### Ethical Review

Prior to submission, this study was licensed with the Chinese Clinical Trial Registry (ChiCTR2000034999). In addition, in accordance with the Declaration of Helsinki, this research was supported by the clinical testing of college, biomedical review board (number: 2020-341) and the Chinese Ethics Committee of Registering Clinical Trials. In addition, the participants signed a formal informed consent form. The research was presented by using the strengthening the reporting of cohort studies in surgery criterion (13).

### Inclusion and Exclusion Criteria

Patients included were those who met the listed inclusion criteria: (1) diagnosed with NSCLC; (2) aged 18–60 years old; (3) undergoing VATS; and (4) complete absence of comorbid conditions such as coronary heart disease and diabetes; (5) the American society of anesthesiologists (ASA) score of 2 or fewer points; and (6) pulmonary nodules not more than 3 cm in diameter.

Exclusion criteria entailed the following: (1) lack of informed consent and (2) patients who had previously received other cancer treatments such as radiotherapy and chemotherapy or a pulmonary surgical procedure.

### Patient Selection and Education

When meeting the inclusion criteria, patients chose to have day surgery (DSG) or inpatient surgery (ISG) according to their own wishes. Two weeks prior to surgery, the patients in the DSG were examined by CT of the head, chest, and upper abdomen, pulmonary function tests, blood tests, blood biochemical tests, and electrocardiography as preoperative evaluations (14). On the day of surgery, the medical experts enlightened the patients with the DSG and their families about the possible risks and complications associated with the procedure and what to expect from it. For the ISG, patients underwent VATS in the same medical group, but in the thoracic surgery ward, which was different from the day surgery center and the preoperative examination and communication of the patients with the ISG were accomplished during the hospitalization days before surgery.

### Surgical Approach

The three-port thoracoscopic technique and double-lumen endotracheal intubation were used to perform the VATS procedure. Intravenous anesthesia was used to sedate the patients. Additionally, there was use of one-lung ventilation (15). The entry point of thoracoscopy was 1.5 cm in the 7th intercostal space just before the midaxillary line. The 3rd and 4th intercostal spaces anterior to the midaxillary line were selected for the main operation port and the 9th intercostal space behind the axillary line was the auxiliary operation port. In this study, systemic dissection of the lymph nodes was performed on all the patients in both the groups.

### Enhanced Recovery After Surgery Program Management

#### Perioperative Fluid Management

Fluid management ensures that patients are not dehydrated before, during, and after surgical operations (16, 17). Prior to the induction of anesthesia, the patients were encouraged to practice carbohydrate loading (18). Balanced crystalloids at an approximate volume of 1–2 ml/kg/h were used for intravenous fluid therapy pre- and postsurgery. The positive liquid balance was maintained at <1,500 ml (or 20 ml/kg/24 h) during the perioperative period (19).

#### Chest Drainage Management

**Figure 1:**
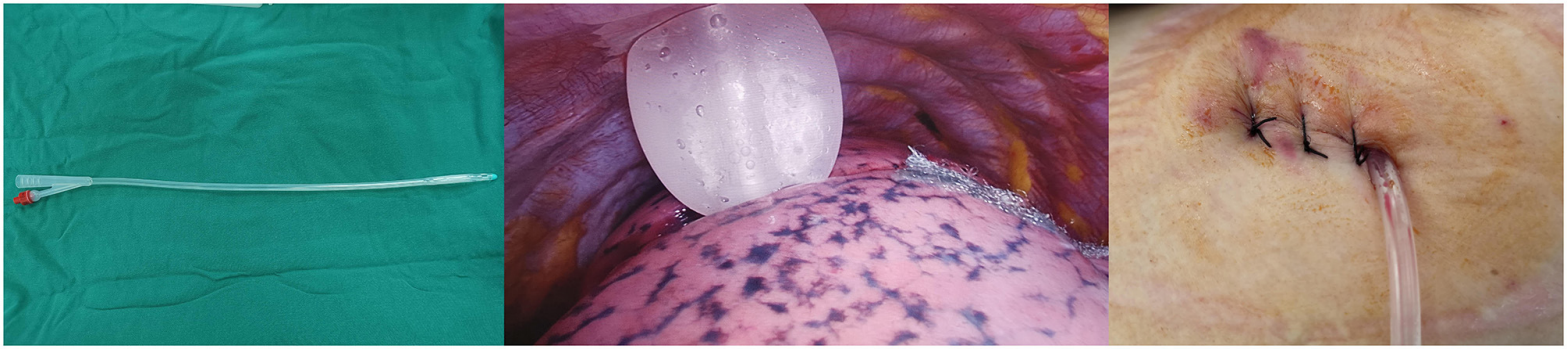
An 18F silicone Foley catheter (filled with 15 ml sterile water) was inserted through a port wound in the 3rd or 4th intercostal middle axillary line and then descended toward the dorsal region.

To drain fluid and air from the pleural cavity, surgery was conducted prior to single chest tube drainage (20). It involved the insertion of a small silicone Foley catheter (18 F), which went through the main operation port and descended toward the dorsal region (Figure 1). Four hours after surgery, once the patients regained consciousness, a chest X-ray was performed to examine whether the lungs remained expanded. The chest tube was removed after confirming the well-being of the lungs and the absence of air leaks. If the daily serous effusion was of high volume (up to 450 ml/24 h), chest tube removal was not performed (21).

#### No Catheterization Management

Before surgery, the patients were told to empty their bladders. During the procedure, insertion of urinary catheters was prohibited (22). Postoperatively, in cases in which the patients had difficulty in urinating, they were presented with optional methods such as adopting a semirecumbent posture and applying warm compression and vulvar rinsing.

#### Regional Anesthesia and Pain Relief

**Figure 2:**
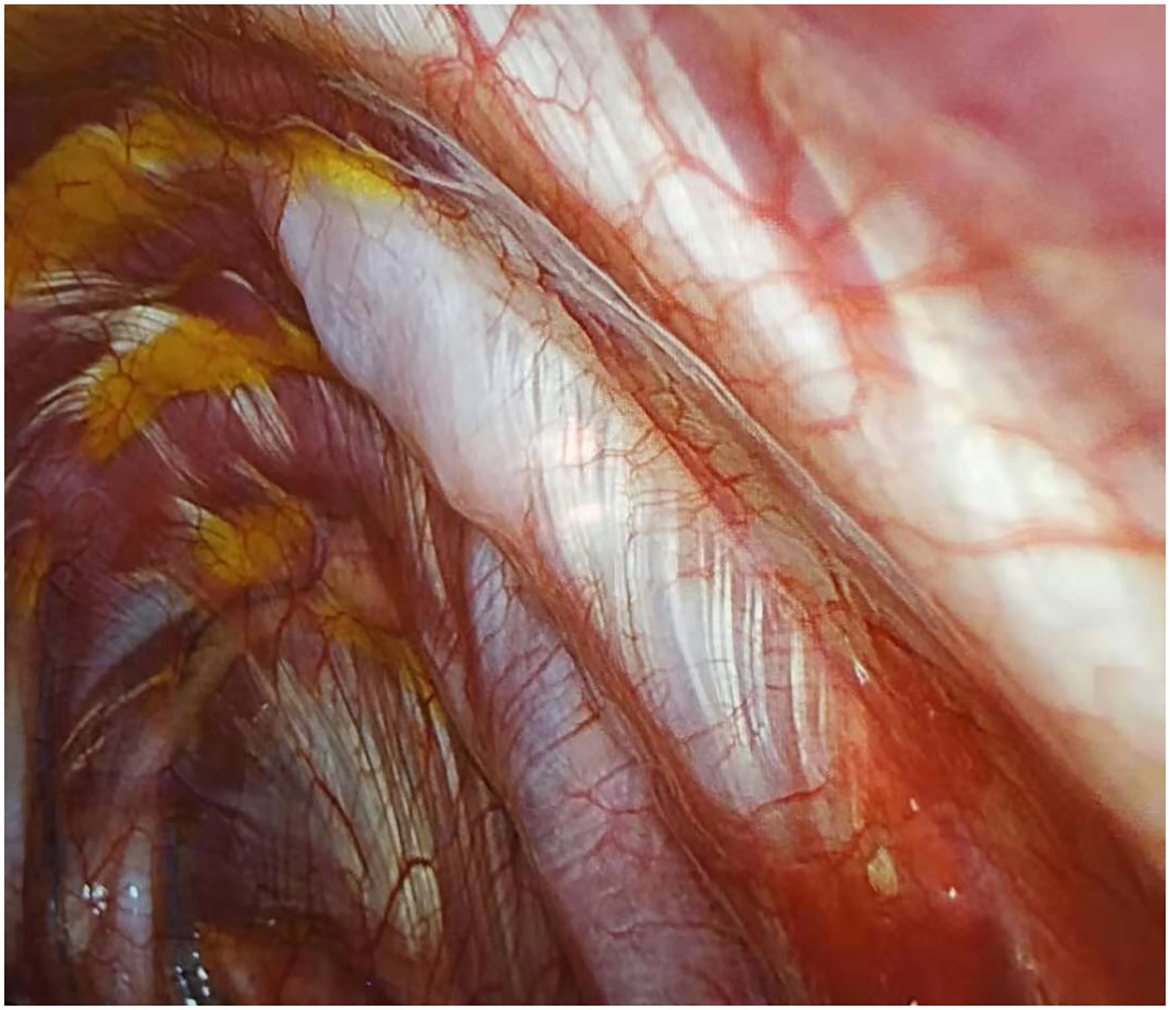
Infiltration of local anesthetic mixture (15 ml for each intercostal space) through thoracoscopic intercostal nerve block (TINB).

The medical team questioned the patients regarding any drug allergies. To induce pre-emptive anesthesia, the patients were injected with parecoxib sodium (Pharmacia & Upjohn Company LLC, Ramsgate Road, England) 1 h prior to the procedure (23). After the completion of the surgery, a local anesthetic mixture was used to infiltrate starting from the 3rd−9th intercostal nerves (Figure 2) by thoracoscopic intercostal nerve blocks (TINBs) (24). Returning to the normal ward 8 h after surgery, the patients were injected with 40 mg of parecoxib sodium (40 mg) (25). Non-steroidal anti-inflammatory drugs (NSAIDs) and ibuprofen capsules were given to the patients if needed.

#### Medium-Chain Triglyceride (MCT) Treatment

Patients were given an MCT diet after the surgical procedure (26, 27). Four hours postoperatively, once conscious, the patients drank warm water (100 ml). After 6–8 h, if the patient showed no sign of vomiting or nausea, the patient drank an appetizing liquid (250 ml). This drink helped with gastrointestinal function recovery. Ten hours postsurgery, patients were supposed to take 50 g of nutritional powder and half a liter of warm water.

### Discharge Criterion

The discharge criteria were categorized into five categories: (1) Certain signs, blood pressure, and pulse; (2) Mental stability; (3) Existence of morning sickness and vomiting; (4) Presence or absence of surgical bleeding; and (5) Presence or absence of pain. To evaluate whether a patient was fit to be discharged, the medical team applied the Postanesthetic Discharge Scoring System (PADSS). For patients to be discharged, they had to score 9 or higher (28).

### Postdischarge Management

To ensure the well-being of the patients postsurgery, the medical officers carried out follow-up communication with the patients and their families. For the 1st week, nurses made phone calls to the patients daily. Follow-up telephone calls were also conducted on the 14 and 28th days after the operation. This procedure was important, since it ensured that patients who experienced any form of medical emergency or those who suffered from any complications from the procedure received immediate medical help. The families were also given training on how to discover surgical bleeding or other complications after going home in time such as observing the breathing rate of the patient and measuring the blood pressure and heart rate of the patient. In cases of severe complications, ambulances were provided to take patients to the emergency wing, surgeons were asked to attend to the patient, and, later, the patient was admitted for further medical assistance.

### Management in the ISG

Because patients in the ISG were in the same medical group as those in the DSG, most ERAS management was implemented in both the groups, but patients in the ISG were admitted before surgery because of relevant preoperative tests and the timing of chest tube removal was the 1st day after surgery when the patients met the chest tube removal standard.

### Endpoints for the Study

The primary outcome was the postoperative outcome of day surgery in patients with NSCLC, which mainly includes: (1) 24-h discharge rate; (2) transfer to hospital rate; and (3) readmission rate within 30 days.

Within 1 month, if any postoperative complications (PPCs) were identified, then they were recorded as the secondary endpoints for this study and they mainly included: (1) pneumothorax: chest X-ray showing that pleural space is >30% and the chest tube is placed again; (2) pleural effusion: chest radiograph shows medium to large effusion; (3) bleeding: more than 200 ml/h of postoperative bloody drainage fluid that lasts for 3 h; (4) hoarseness; (5) pulmonary infection: clear etiological evidence, imaging showing atelectasis or large patches, fever, and total number of white blood cells >10,000/mL; (6) prolonged air leak (PAL): air leak that persists for more than 5 days postoperatively; and (7) chylothorax: chylous test (+) and daily drainage volume >500 ml.

In addition, the treatment-related costs and resource consumption were also recorded, which included: (1) average hospital cost incurred during hospitalization not including expenses incurred in outpatient examinations or treatment; (2) drug costs and material costs; (3) chest drainage tube retention time and drainage volume, including the period of chest drain (h) and drainage volume per hour (mL/h); and (4) LOS after surgery.

### Statistical Analysis

Propensity score matching was used to compare characteristics between the two distinctive cohorts by using observational data from the hospital information system. The regression model [dependent variable was management (day surgery or inpatient surgery) and the independent variables were preoperative factors of the patient (sex, age, pulmonary function, smoking history, and body mass index (BMI)] and hospital characteristics (operation method, tumor node metastasis (TNM) stage, and pathology) were used to calculate PSM for every patient. PSM for discharges was also calculated by using the regression model. Within the two study cohorts, discharges were randomly sorted. Then, using the closest PSM, each discharge in the DSG was matched 1:2 to a discharge in the ISG. The balance of measured covariates was assessed by using the *p*-value with ≥ 0.05 representing a significant difference between the study groups.

Demographic data collected were recorded as follows: the means and SDs represented continuous data, medians and ranges represented non-normally distributed data, and proportions were represented by binary variables. The Student's *t*-test and the Mann–Whitney *U*-test were used to make comparisons. In the case of categorical data, the chi-squared test or the Fisher's exact test was used to perform the comparisons. *P* < 0.05 (two-tailed) was found to be statistically significant in all of the analyses. All the statistical analyses were conducted by using the Statistical Package for the Social Sciences (SPSS) software (version 22.0, IBM Corporation, Armonk, New York, USA), which was used to analyze the data.

## Results

### Study Population

**Figure 3:**
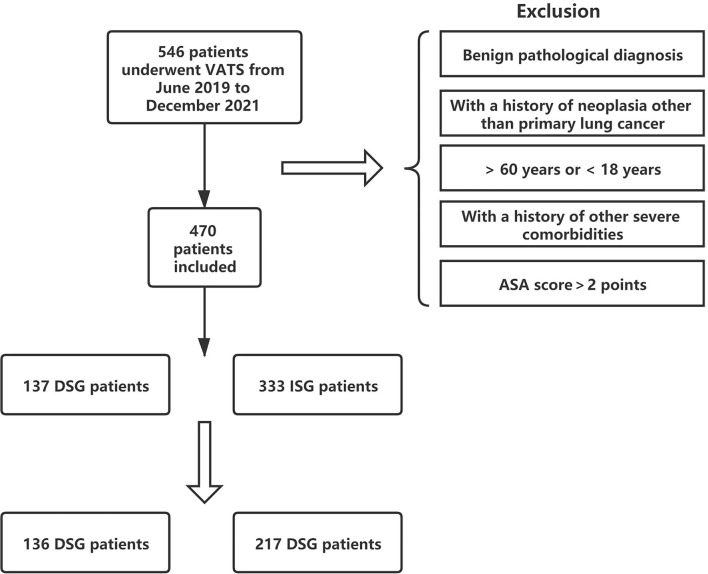
Study flowchat.

**Figure 4:**
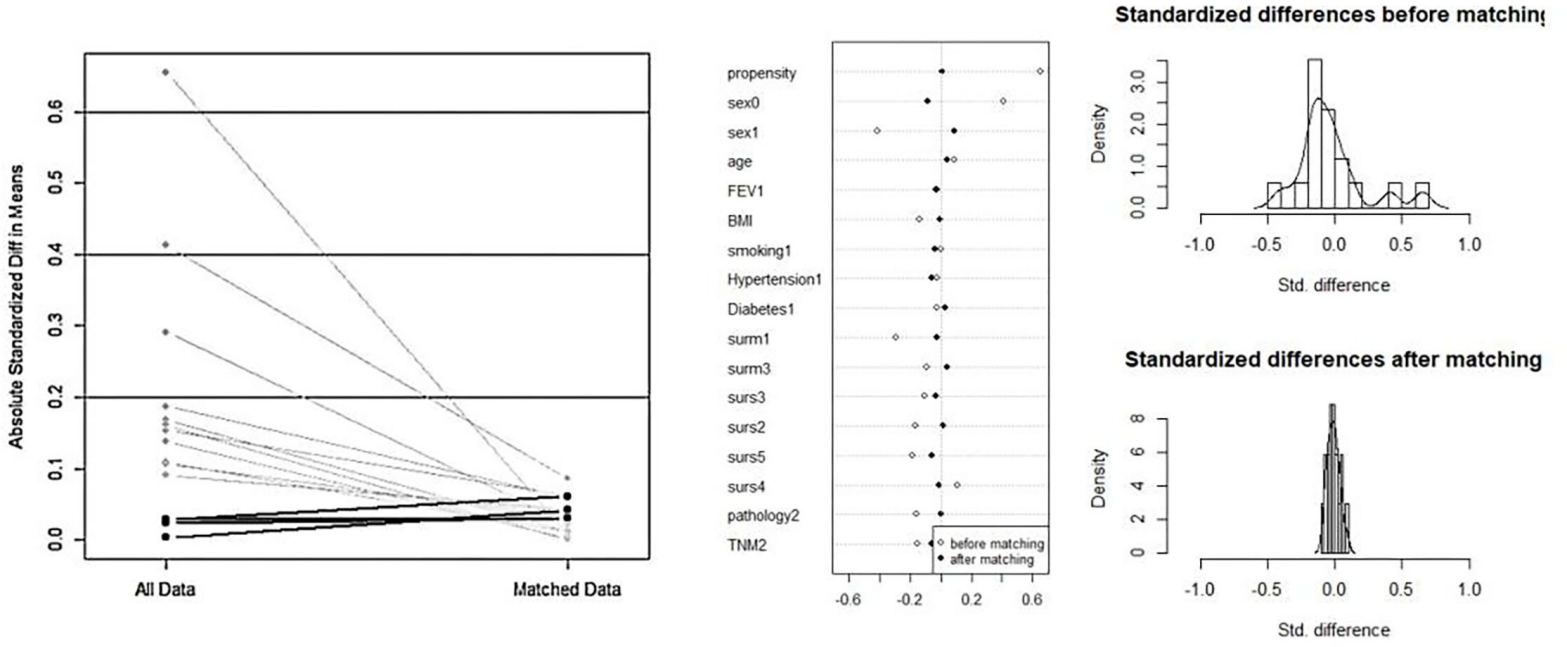
The process of propensity score matching (PSM). After PSM, the clinical characteristics and baseline data between the two groups are matched and comparable.

Overall, 470 patients underwent VATS for NSCLC in the same medical group from June 2019 to December 2020. Among them, 137 (29.1%) patients were in the DSG and 333 (70.9%) patients were in the ISG (Figure 3). Table 1 presents the characteristics of patients in the DSG and ISG before and after PSM (details can be seen in Figure 4). After PSM, 353 patients in total were enrolled in this study including 136 patients in the DSG and 217 patients in the ISG. The clinical characteristics, pulmonary function, surgical procedures, histology, and TNM stage [2017 union for international cancer control (UICC)], which were matched and comparable, are listed in Table 1.

**Table 1:** Population characteristics of in two groups before and after propensity score matching.

**Index**		**Overall cohort**			**Matched cohort**		
		**DSG** **(***n*** = 137)**	**ISG** **(***n*** = 333)**	***p*** **-value**	**DSG** **(***n*** = 136)**	**ISG** **(***n*** = 217)**	***p*** **-value**
Gender	Male	25	114	0.001	25	40	0.990
	Female	112	219		111	177	
Age (year)		43.36 ± 9.25	42.53 ± 10.25	0.407	43.30 ± 9.26	42.76 ± 10.66	0.629
BMI		23.00 ± 2.59	23.35 ± 2.91	0.212	22.99 ± 2.60	23.15 ± 2.88	0.616
Smoking	Yes	10	24	0.972	10	18	0.750
	No	127	209		126	199	
Pulmonary function	FEV1, L	3.02 ± 0.74	3.04 ± 0.73	0.819	3.02 ± 0.74	3.03 ± 0.76	0.920
	FEV1/FVC, %	82.84 ± 7.58	82.87 ± 7.67	0.970	82.84 ± 7.58	82.85 ± 7.46	0.984
Comorbidities	COPD	0	0	1.000	0	0	1.00
	Hypertension	2	6	0.795	2	5	0.585
	Diabetes	2	6	0.795	2	3	0.946
Operation approach	Lobectomy	51	160	0.008	51	92	0.351
	Segmentectomy	85	168		84	124	
	Wedge resection	1	5		1	1	
Surgical site	LUL	49	76	0.039	48	63	0.266
	LLL	16	57		16	28	
	RUL	43	121		43	75	
	RML	14	23		14	20	
	RLL	15	56		15	31	
Histology	Adenocarcinoma	134	326	0.296	135	215	0.853
	Squamouscarinoma	3	7		1	2	
TNM stage (2017 UICC)	I	135	322	0.269	134	211	0.427
	II	2	11		2	6	
	III or IV	0	0		0	0	

*DSG, day surgery group; ISG, inpatient surgery group; FEV1, forced expiratory volume in1s; FVC, forced vital capacity; COPD, chronic obstructive pulmonary disease; LUL, left upper lobe; LLL, left lower lobe; RUL, right upper lobe; RML, right middle lobe; RLL, right lower lobe*.

### Outcomes of Day Surgery Patients With Lung Cancer

**Figure 5:**
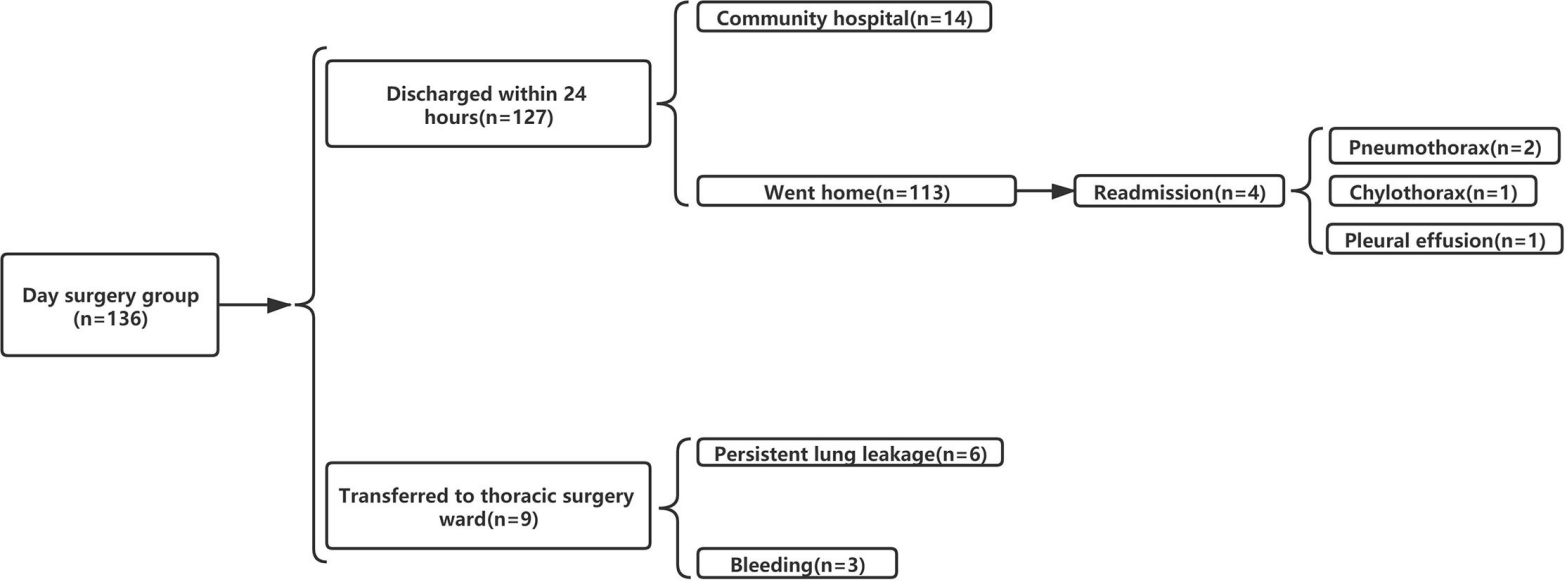
Outcome in day surgery group (DSG).

Among 136 patients with NSCLC undergoing VATDS, the 24-h discharge rate was 93.38% (127/136). Among the 127 patients who were discharged, 113 patients returned home after discharge from the day surgery center and underwent rehabilitation training at home and 14 patients went to community hospitals for rehabilitation training with the help of therapists and returned home soon thereafter within 1 or 2 days. A total of nine individuals were admitted to the thoracic surgery ward from the day surgery center because of PPCs of whom six patients had PAL and three patients had postoperative intrathoracic hemorrhage. The six air leakage patients were treated with chest drains and the median LOS of these patients in the thoracic surgery ward was 5 days. All of the patients had recovered well by the follow-up visit. Among the three postsurgery patients who experienced bleeding, one had to undergo thoracoscopic hemostasis surgery and was later taken to the thoracic ward. The remaining individuals were also moved after using hemostatics. Three patients whose vital signs had recovered after treatment were discharged after being observed for some time. The rate of readmission within 30 days was 2.94% (4/136). Two patients who had severe dyspnea after discharge were readmitted because of pneumothorax diagnosed by chest X-ray and they underwent thoracic drainage for 3 days in the thoracic surgery ward. One patient received thoracentesis in the emergency department due to chest tightness and shortness of breath 12 days after the operation. When diagnosed with chylothorax, she was admitted to the hospital for conservative treatment and discharged after 1 week. One patient experienced medium pleural effusion diagnosed by chest X-ray and underwent thoracentesis in the emergency department. Then, she was treated in the hospital for 3 days and discharged after the volume of chest drainage was <50 ml/day. Details can be seen in Figure 5.

### Postoperative Complications Between the Two Groups

No significant difference was found between the DSG (11.76%) and the ISG (11.52%) (*p* = 0.944) with respect to the PPCs. The incidences of bleeding, hoarseness, and pulmonary infection in the DSG (2.21, 1.47, and 0.74%) were high, but there were no statistically significant differences compared with the ISG (1.84, 1.38, and 0.00%) (*p* = 0.579, *p* = 0.757, and *p* = 0.206). The difference in the incidence of chylothorax between the DSG (0.74%) and the ISG (0.92%) (*p* = 0.853) was insignificant. The incidences of PAL, pneumothorax, and pleural effusion were higher in the ISG (4.61, 1.84, and 0.92%) than in the DSG (4.41, 1.47, and 0.74%) and there were insignificant differences (*p* = 0.931, *p* = 0.792, and *p* = 0.853). Details can be seen in Table 2.

**Table 2:** The comparision of postoperative complications between the two groups.

**Index**	**DSG** **(***n*** = 136)**	**ISG** **(***n*** = 217)**	***P***
PAL	6 (4.41%)	10 (4.61%)	0.931
Pneumothorax	2 (1.47%)	4 (1.84%)	0.792
Pleural effusion	1 (0.74%)	2 (0.92%)	0.853
Hoarseness	2 (1.47%)	3 (1.38%)	0.946
Bleeding	3 (2.21%)	4 (1.84%)	0.812
Chylothorax	1 (0.74%)	2 (0.92%)	0.853
Pulmonary infection	1 (0.74%)	0 (0.00%)	0.206
Total	16 (11.76%)	25 (11.52%)	0.944

*PAL, prolonged air leakage*.

### Treatment-Related Costs and Resource Consumption

From Table 3, it is obvious that a shorter mean LOS after surgery (1.47 ± 1.09 vs. 2.72 ± 1.28 day, *p* < 0.001) was observed in the DSG than in the ISG. Patients in the DSG had a significantly shorter drainage duration than controls (8.45 ± 3.35 vs. 24.11 ± 5.23 h, *p* < 0.001); however, drainage volumes per hour were not significantly different between groups (19.52 ± 5.95 vs. 20.15 ± 5.42 mL/h, *p* = 0.312). There was no difference observed in the cost of equipment and materials between the two groups (3,424.52 ± 448.47 vs. 3,375.29 ± 473.40 $, *p* = 0.333). However, the average hospital cost in the DSG was significantly lower than that in the ISG (6,411.47 ± 657.76 vs. 7,522.41 ± 1,471.84 $, *p* < 0.001). A lower average drug cost was found in the DSG than in the ISG (274.20 ± 63.67 vs. 622.29 ± 253.68 $, *p* < 0.001). Details can be seen in Table 3.

**Table 3:** Treatment-related costs and resource consumption between two groups of patients.

**Index**	**DSG** **(***n*** = 136)**	**ISG** **(***n*** = 217)**	***p***
LOS (d) (after surgery)	1.47 ± 1.09	2.72 ± 1.28	0.000
Period of chest drain (h)	8.45 ± 3.35	24.11 ± 5.23	0.000
Drainage volume per hour (mL/h)	19.52 ± 5.95	20.15 ± 5.42	0.312
Average hospital cost (USD)	6,411.47 ± 657.76	7,522.41 ± 1,471.84	0.000
Drug cost (USD)	274.20 ± 63.67	622.29 ± 253.68	0.000
Materials cost (USD)	3,424.52 ± 448.47	3,375.29 ± 473.40	0.333

*LOS, length of stay; USD, USA dollar*.

## Discussion

**Figure 6:**
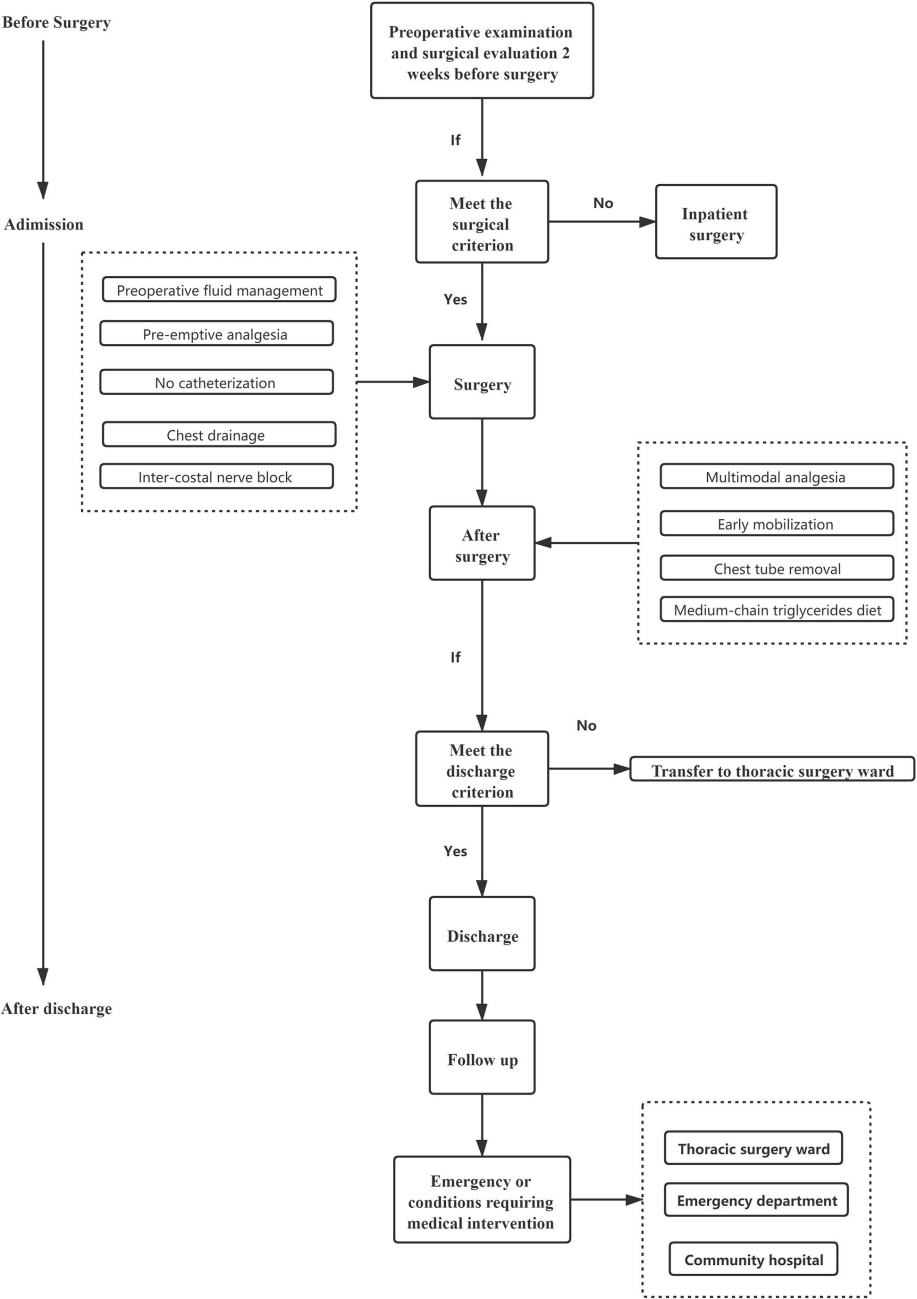
Clinical pathway management of video-assisted thoracoscopic day surgery (VATDS).

The clinical pathway management of VATDS was shown in Figure 6. This study suggests that VATDS is a safe and feasible technique for a highly selected group of patients with early-stage NSCLC. Patients in the DSG had an acceptable discharge rate within 24 h (93.38%) and those patients who had not been discharged due to PPCs (six patients for air leakage and three patients for bleeding) were also discharged after symptomatic treatment. Compared with the ISG, the DSG showed no significant difference in the rate of PPCs. In addition, because of earlier chest tube removal, the DSG had a shorter LOS after surgery and lower hospitalization costs than the ISG. However, there are needs to select the patients for day surgery and for further discussion concerning the large-scale use of VATDS.

The successful implementation of VATDS cannot be separated from the participation of ERAS management and minimally invasive technology. In the recent years, the promotion of ERAS and minimally invasive surgery has aimed to help minimize perioperative stress and morbidity rates and to expedite postsurgery recovery (29–34). In this study, the patients in the DSG and ISG were from the same medical group in which ERAS management was fully employed. Pain control, single small chest tube drainage, no indwelling urinary catheter, and an MCT diet are the main components of our ERAS programs. Pain control is one of the main issues in ERAS programs (35). Currently, instead of patient-controlled analgesia (PCA) only, TINBs, together with a postoperative combination of acetaminophen and NSAIDs, are used in our hospital. Most of the patients in this study reported that the pain was relieved and the comfort level was obviously improved, which is a very important cause of early discharge. In the ERAS program, single small chest tube drainage was performed in both the groups. To expel gas and fluid from the thoracic cavity to prevent infection, an 18F silicone Foley catheter thoracic drainage tube was installed after surgery (27). A small chest tube provides better comfort for the patients under the precondition of no increase in PPCs, which, together with early chest tube removal, makes the discharge of the patient on the first day after surgery feasible. With the use and advancement of ERAS in the presurgery period, the lack of an indwelling urinary catheter plays an important role in the tubeless process of VATS, especially in the DSG. Both groups in this study had no patients without urinary catheters presenting postoperative urinary retention or urinary tract infection after the surgery. We also paid attention to the nutritional status of the patients in our ERAS management. For example, glucose liquid is given to the patients before surgery and special soups are prepared for the patients 2 h after surgery, which can accelerate the recovery of gastrointestinal function and improve dizziness symptoms. Thus, the improvement of the ERAS program in thoracic surgery has led to better recovery and satisfaction of the patients. In West China Hospital, the enhanced recovery after thoracic surgery (ERATS) program has been implemented for many years and its advantages are obvious (36).

Day surgery is welcomed because of many aspects and modern ERATS could make VATDS possible. In addition to the implementation of an ERAS program for in-patient surgical care, the patients in the DSG underwent preoperative examinations in an outpatient center 2 weeks before surgery, which is more convenient than in-hospital examination and maximizes the utilization of limited medical resources. In addition, chest tubes of the patients with the DSG were removed earlier than those of the ISG, which is feasible and did not increase the rate of PPCs such as pleural effusion or pneumothorax. Early removal of the chest tube reduces postoperative pain, reduces the LOS, and potentially decreases in-hospital costs. In this study, the patients in the DSG spent less money than those in the ISG. We could attribute this outcome to the shorter duration of LOS, which led to lower costs of hospitalization per day and fewer medical interventions such as atomization inhalation and chest drainage nursing, which were used as routine treatments for postoperative days in the patients with the ISG but not in the patients with the DSG.

In this study, among the 136 outpatient surgery patients, 93.38% of the patients were discharged within 24 h. The remaining 6.62% of the patients were not discharged until further discussion. A total of six patients who were not discharged had air leakage, while three patients experienced postoperative bleeding. One of the major PPCs after lung surgery is prolonged air leakage (37). According to studies conducted in the past (38, 39), the occurrence of PAL was 10%. Among the six patients who had air leakage, five patients underwent right-side lobectomy; in three patients of them, fissures did not develop, while two patients suffered from pleural adhesion, which could contribute to PAL. An ERAS group from Italy (40) pointed out that one of the crucial parts of ERAS was the prevention of air leakage and measures such as pleural tent and staple-line reinforcement should be performed in the high-risk patients. Mostly, in day surgery centers, air leakage limits the discharge of day surgery patients. Therefore, it is necessary to develop a prevention approach for air leakage. Additionally, the safety of discharge with a chest tube after pulmonary segmentectomy in the selected patients has been reported (Bao) (41). There is a need to further confirm the validity of this approach and it would be of high importance in VATDS management. In addition to making VATDS possible, complete clinical pathway management should be performed to ensure the safety of patients after surgery as shown in Figure 1. A total of 14 patients without any symptoms went to community hospitals for better recovery, which was much more than the number of the patients with the ISG. Therefore, we must perform better and more comprehensive preoperative education to eliminate or lessen the concerns of the patients. Moreover, the “two-way referral policy” might need to be completed. The Chinese government encourages high-level hospitals to refer the patients with less serious conditions to primary hospitals, while primary hospitals refer the patients with more serious conditions to higher-level hospitals based on the Chinese population and medical background. Therefore, for those patients who cannot rest well at home or who are worried about their condition after surgery transfer to primary hospitals might be needed.

Considering the large population of China, medical resources should be maximally used. To minimize medical pressure in China, it might be wise to approach the patients with NSCLC who are still in the early stage and with few or no conditions. However, VATDS practice is still being explored; thus, it might be impossible to use it in all the selected patients who meet the inclusion criteria. Of the 136 patients with early-stage NSCLC undergoing outpatient surgery, 16 patients were found to have postsurgery complications: six patients had PAL, two patients had contracted pneumothorax, three patients suffered from hemorrhages after the procedure, two patients had hoarseness after surgery, and three patients had pleural effusion and pulmonary infection. These rates were not significantly different compared to the patients in the in-hospital group. Thus, we could say that VATDS was well-established by the ERAS program and strict patient selection and was not implemented at the expense of patient safety.

According to the findings of this study, we can say that VATDS is uncommon and few studies have reported this fact (9–11). However, ERAS management combined with minimally invasive surgical techniques has rendered the conversion of many surgeries in different specialties from inpatient to day surgery successful. Vendittoli et al. (42) reported that implementation of an ERAS short-stay protocol (LOS < 24 h) for patients undergoing hip or knee joint replacement resulted not only in reduced hospital LOS, but also in improved patient care and reduced direct healthcare costs. Dumestre et al. (43) demonstrated that same-day surgery and discharge for eligible patients who underwent alloplastic breast reconstruction were safe and feasible when conducting an ERAS protocol. By reducing pain while minimizing opioid use and its side effects, improving patient function and early activity, improving perioperative bowel function, reducing wound complications, and ultimately reducing the risk of deep vein thrombosis, ERAS is used to improve patient recovery to a level at which the patient will be able to leave the hospital sooner. Under this condition, VATDS could be implemented *via* ERAS. In addition, VATS has already proved to have a lower lung infection rate, less pain, and shorter postoperative LOS than open lobectomy (44, 45), which will be a desirable surgical approach for VATDS. Moreover, Cui (46) and his group reported on the safety and feasibility of tubeless VATS under non-intubated, intravenous anesthesia with spontaneous ventilation, and no placement of chest tubes postoperatively and it had certain advantages in selected patients with thoracic disease. Although this approach was not conducted in this study, it could be a further study direction for VATDS.

## Limitations

Some limitations of this study cannot be overlooked. All of the patients were enrolled in the program from June 2019 to December 2020 in the same medical group in a single regional center. First, this study was not a randomized controlled trial; therefore, it might be affected by other factors. Second, the relevant conclusion of this study cannot be generalized because the patients selected were those receiving VATS for pulmonary nodules. Furthermore, we only compared the patients in two groups after strict selection; a wider scope of patient selection and reduced inclusion criteria should be included in further studies. Finally, the follow-up period should be extended with a more comprehensive assessment. By doing so, it will ensure that the viability and safety of VATDS are correctly captured.

## Conclusion

This study indicates that individuals who underwent VATDS had an acceptable discharge rate within 24 h and that day surgery vs. inpatient surgery was associated with no significant differences in PPCs with a shorter mean duration of LOS after surgery, shorter drainage duration, and lower hospital costs. However, it is necessary to select patients for VATDS. Additionally, there should be further discussions regarding the large scale use of VATDS.

## Data Availability Statement

The original contributions presented in the study are included in the article/supplementary material, further inquiries can be directed to the corresponding author/s.

## Ethics Statement

The studies involving human participants were reviewed and approved by the University's Clinical Trials and Biomedical Ethics Committee (Number: 2020-341) and the Chinese Ethics Committee of Registering Clinical Trials. The patients/participants provided their written informed consent to participate in this study.

## Author Contributions

YD: conceptualization, writing-original draft, and software. CS: data curation, methodology, and preparation. YW: visualization and investigation. KZ: writing—reviewing and editing and supervision. JL: software and validation. SC: formal analysis. HM: conceptualization. GC: supervision and conceptualization. All authors contributed to the article and approved the submitted version.

## Funding

The National Natural Science Foundation of China (No. 72104161) sponsored this study.

## Conflict of Interest

The authors declare that the research was conducted in the absence of any commercial or financial relationships that could be construed as a potential conflict of interest.

## Publisher's Note

All claims expressed in this article are solely those of the authors and do not necessarily represent those of their affiliated organizations, or those of the publisher, the editors and the reviewers. Any product that may be evaluated in this article, or claim that may be made by its manufacturer, is not guaranteed or endorsed by the publisher.
